# Adolescents with developmental traumas in therapy in a child and adolescent mental health service, outpatient unit: experiences of daily living and expectations for therapy – a qualitative study

**DOI:** 10.3389/fpsyg.2023.946394

**Published:** 2023-05-05

**Authors:** Kjerst Olstad, Torgeir Sørensen, Lars Lien, Lars Johan Danbolt

**Affiliations:** ^1^Innlandet Hospital Trust, Brumunddal, Norway; ^2^MF Norwegian School of Theology, Religion and Society, Majorstuen, Oslo, Norway; ^3^Faculty of Health Science, VID Specialized University, Oslo, Norway; ^4^Norwegian National Advisory Unit on Concurrent Substance Abuse and Mental Health Disorders, Hamar, Norway

**Keywords:** developmental trauma disorder, adolescents, self-understandings, therapy, systematic text condensation

## Abstract

**Background:**

Developmental trauma has a profound effect on people’s lives. There are few studies of the perceived difficulties and treatment needs of adolescents with developmental trauma. More studies are called for to explore the perspectives and experiences of these patients, especially adolescents.

**Method:**

Semi-structured interviews were conducted with eight adolescents with developmental trauma aged 14–18 years in a Child and Adolescent Mental Health Service, Outpatient Unit. The interviews were analyzed using systematic text condensation.

**Results:**

A main finding in this study regards the participants’ understanding of why they needed therapy: symptom alleviation and coping tools. They expressed a need for talking with a safe and reliable adult who understood their situation. Their stories of daily functioning and bodily sensations align mainly with symptoms described for adolescents with developmental trauma. The study also shows that the participants’ experiences of trauma affected their lives to a greater or lesser extent, in the form of ambivalence, avoidance, regulation, and coping strategies. They also described various physical problems, especially insomnia and inner restlessness. Their subjective stories revealed insights into their experiences.

**Conclusion:**

Based on the results, we recommend that adolescents with developmental trauma be allowed to express their understandings of their difficulties and their treatment expectations at an early stage of their therapy. An emphasis on patient involvement and the therapeutic relationship can increase their autonomy and control over their own lives and treatment.

## Background

Childhood trauma can disrupt children’s development and in many cases cause lifetime psychological distress (van der Kolk, 2014, pp. 149–150; Felitti et al., 2019; Thoma et al., 2021). In research or clinical contexts, there is a clear correlation between violent childhood experiences and mental and physical health problems, daily functioning, and social relationships (Hughes et al., 2017; Bellis et al., 2019; Rueness et al., 2020; Birkeland et al., 2021). Trauma is often described as developmental trauma when it is related to the child’s experiences. It typically occurs early in life and over the course of development and includes neglect, maltreatment and domestic sexual, physical and emotional abuse, as well as frequent separations or violence involving the child’s caregivers (Nordanger et al., 2011).

The concept of developmental trauma is debated, especially in terms of its inclusion as a diagnosis in the diagnostic manuals (van der Kolk et al., 2009; Cruz et al., 2022). The background is the discussion about the long-term consequences of childhood trauma (Schmid et al., 2013) and whether PTSD is sufficient to describe the symptoms of ACEs (adverse childhood experiences) and the developmental consequences in children and adolescents with persistent, complex trauma experiences (van der Kolk et al., 2009). PTSD is limited in children and adolescents as it is based on the symptom picture in adults. Other diagnostic criteria have therefore been proposed (Scheeringa et al., 2003; Simons and Herpertz-Dahlmann, 2008; Euser et al., 2010; D’Andrea et al., 2012). In the DSM-5, Developmental Trauma Disorder (DTD) has now been included as a new diagnosis. In the ICD-11, complex PTSD (c-PTSD) is a new diagnosis, also validated for adolescents (Kazlauskas et al., 2020). A study using the DTD Structured Interview and Traumatic Events Screening Instrument shows that although PTSD and DTD share traumatic antecedents, DTD is uniquely associated with traumatic emotional neglect/abuse with simultaneous separation of caregivers (Spinazzola et al., 2021). Until now, DTD is not a diagnosis in Norway, and to avoid confusion, we use the abbreviation DT (Cruz et al., 2022) for developmental trauma in this study. The evaluation of DT among the participants in the present study was based on the first assessment made by psychologists, psychiatrist or other qualified personnel at the recruitment clinic during the anamnesis.

In the present study, developmental trauma is understood as a stressful situation where two negative factors, traumatic stress and poor emotional regulatory support, occur simultaneously (Nordanger, 2017). Attachment disorder is often a result of such trauma (Anstorp et al., 2006; Lim et al., 2020). Security, stable care relationships and support from family members or other adults have been highlighted as particularly important factors in prevention and stabilization (Mezzich et al., 2016; Gondek et al., 2017). The symptoms often described as typical of DT, are emotional and psychological dysregulation, physical health problems, disturbed sensory perception, insomnia, self-harm, self-destructive behavior, difficulties with executive functioning and attention regulation, poor self-regulation, and problems with establishing relationships (van der Kolk et al., 2009; Stolbach et al., 2013; Spinazzola et al., 2021).

Adolescence is a vulnerable stage of life with a high risk of developing mental health problems (Kessler et al., 2007; Jones, 2013). Both in Norway and internationally, mental health challenges in adolescents are on the rise (Sletten and Bakken, 2016). Child and adolescent mental health services provide assessment and treatment until the age of 18 in Norway. In 2019, more than 56,000 patients were treated in Child and Adolescent Mental Health Services, Outpatient Units (CAMHS) in Norway (Norwegian Directorate of Health, 2019). There are no figures for developmental trauma as this is not a diagnostic term recorded in patient registers.

The introduction of care pathways in 2019 aimed to ensure equal and coordinated treatment with increased patient involvement, but experience to date shows little success in this area (Ådnanes et al., 2020). There is a call for more knowledge of patient perceptions of treatment quality and greater influence of adolescents on mental healthcare services (Norwegian Ministry of Health and Care Services, 2019).

The patient perspective is more strongly emphasized in an approach that assumes that patients, with their resources, experiences, wishes, and goals, should be at the center of integrated care and treatment (Mezzich et al., 2016; Gondek et al., 2017; Birkeland et al., 2022). Emphasis is placed on co-determination in all treatment decisions (Ekman et al., 2014) in line with patient-centered health policy guidelines (Norwegian Ministry of Health and Care Services, 2017; Norwegian Directorate of Health, 2019).

We know a great deal about the symptoms children develop following early childhood trauma, but less about their later experiences, and recent research has delved deeper into the consequences of trauma, particularly patients’ subjective experiences (Danese, 2020; Smith and Pollak, 2021). However, a systematic literature search revealed few studies of adolescents with developmental trauma and their own understanding and experience of their situation when they are in therapy in a mental health clinic for children and adolescents. In treatment for eating disorders, substance abuse, anxiety and depression, as well as various phobias in children with high-functioning autism, research has shown that patients’ motivation and understandings are important factors in helping them benefit from treatment (Zuroff et al., 2007; Buckner et al., 2008; Medalia and Saperstein, 2011; Allen et al., 2012; Karnezi and Tierney, 2021; Lassen et al., 2022).

A Norwegian study of young people who had experienced at least one traumatic event (e.g., sexual abuse, domestic violence, peer violence, life-threatening accidents, or the sudden death of a parent) who received trauma-focused cognitive therapy, showed that few or none of them expected the therapy to help them and that talking about the trauma was difficult even though they found it useful (Dittmann and Jensen, 2014). The study also showed that the patients were happy to talk to therapists because of their expertise, neutrality and confidentiality. They also found it useful to learn skills to reduce stress. Qualitative studies have also been conducted that show young people’s experiences with a specific treatment program such as multisystemic therapy (MST; Tighe et al., 2012; Paradisopoulos et al., 2015; Bunting et al., 2021). However, a literature review (De Haan et al., 2013) concludes that studies on therapy rarely investigate adolescent patients’ subjective experiences and calls for studies that focus on these patient perspectives. Our interest in the present study is limited to the adolescents’ own reflections on why they need therapy, not on their experiences with therapy during or after periods of treatment. Their need for therapy concerns their subjective assessment of their situation and what they expect to gain from CAMHS therapy. Daily life functioning includes behavioral as well as bodily experiences.

Against this background, the overall purpose of the present study was to explore how adolescents with developmental trauma describe and reflect on their need for therapy and their experiences of daily life functioning. The following two research questions were raised:

- How did adolescents with symptoms of developmental trauma describe and reflect on their need for therapy in CAMHS?
- How did these adolescents describe and reflect on their daily life functioning?

## Materials and methods

### Design and setting

This study has a qualitative exploratory research design based on semi-structured in-depth interviews. This provides flexibility to capture nuances and particular features of a field about which there is little previous knowledge (Braun and Clarke, 2013). The design is suitable for gaining new knowledge about the experiences of a group of people (Bate and Robert, 2007).

### Participants and recruitment

The interviews were conducted with eight adolescents with developmental trauma in therapy in a CAMHS. In accordance with criteria for information power and sample size in qualitative research, we considered that around eight participants would provide good information (Malterud et al., 2016). For reasons of privacy, we do not provide specific information about individual participants, only an overall picture of who they were. We contacted the unit manager of a CAMHS in a small town in Norway to provide oral and written information about the study. The inclusion criteria were 14- to 18-year-old and assessed with DT during psychological anamnesis. Those in active substance use or psychosis were excluded. The therapists were asked to assess whether there was concurrent traumatic stress and poor regulatory support and the adolescents’ suitability for participation on the basis of the referral and screening interview. An important factor was whether they had mild enough symptoms to cope with an interview. This study does not describe the participants’ diagnosis or treatment approach. However, they all had developmental trauma as a complex backdrop.

The participants were all in an early stage of outpatient treatment for their trauma at a CAMHS in Norway, and they were all receiving the same treatment. We do not have information about any medication given to the participants as a possible adjunct treatment to psychological therapy. The following types of traumatic experiences were mentioned: a high level of family conflict, rape, several broken relationships, violence, violations, and parental alcohol and drug use. The common feature was traumatic stress coupled with simultaneously poor emotional regulatory support from caregivers. The participants were born and grew up in Norway and were attending school. At the time of the interview, five of them lived at home with their mother, father or both parents. One lived with a boyfriend, while two lived in foster homes. Two had been inpatients in a psychiatric ward for adolescents. Most had moved several times, either with their family or to various forms of emergency accommodation before moving to a foster home or back to their parents’ home. Several of them reported having had quite strong suicidal thoughts, but none were considered by the therapists to be at risk of suicide during the period of the interviews. Although an even gender distribution was sought, only one participant was male.

### Data collection

An interview guide with open, thematic questions was used. Specificity was high, but there was also room for the participants’ own stories, whereby we aimed to obtain data with high information power, which in qualitative research does not solely depend on the sample size, but on such factors as the quality of the research questions, sample specificity, theoretical anchoring, interview dialog and analysis strategy (Malterud et al., 2016). The interviews were conducted by the first author from October 2019 to August 2020. To prevent the interview situation from being too overwhelming for the participants, we decided that only one researcher would conduct the interviews. The interviews were discussed by the entire research team shortly afterward with a view to capturing immediate impressions. Interviews lasted from 30 to 60 min.

In line with the aim of the study, the following four questions in the interview guide were used as a checklist to keep the conversation to their narrative about their experiences, where the first two items refer to need for therapy and the last two concern daily functioning:

1. *Why are you being treated at this clinic?*
2. *How do you understand your difficulties or challenges?*
3. *What is your life like these days?*
4. *How does your body feel after your trauma or traumas?*

The question about their bodily experiences was specified and included after the second interview because it appeared to be important in the two first interviews regarding daily life experiences, and we wanted to ensure that this perspective was also included in the rest of the interviews. Bodily experiences among traumatized adolescents is a prominent topic in the literature (Siegel, 2003; van der Kolk, 2014; Spinazzola et al., 2021). The rationale for these four questions was then to have a person-centered approach in order to explore the participants’ subjective experiences and reflections and still keep the focus of the aim.

Five interviews took place in a CAMHS clinic. The last three interviews were conducted by telephone due to the COVID-19 situation.

### Data analysis

A separate analysis was conducted for each of the two parts of the aim, how adolescents with developmental trauma describe and reflect on (a) their need for therapy and (b) their experiences of daily life functioning. This means that the main categories were theory-informed, but the responses to each of the questions were inductively analyzed using systematic text condensation (STC), a cross-case thematic analysis for qualitative data especially developed for health research and well suited when the aim is to explore meaning and content of data across cases (Malterud, 2012). The analysis was performed in four steps: (1) First, we read through the entire material with an open mind to gain a general idea of it and identify preliminary themes. (2) We then read the text closely to identify and code meaning units that represented the participants’ experiences and reflections. A meaning unit is a text fragment containing some information about the research question. (3) Next, we summarized and condensed the content of each code group and sorted the different meaning units in each group into subgroups (condensates). During this process, we identified suitable quotes from the material that would illustrate the themes. (4) Finally, an objectified summary description of the content was created, illustrated with individual stories and quotations typical of the topics and findings of the study (Malterud, 2012).

To ensure rigor, we followed the procedural standards of systematic text condensation, and all four authors participated in the steps of the analysis, and all critically reviewed and discussed the interpretation of the results (Malterud, 2012; Malterud, 2017). Before each interview, the previous interview was read through in order to plan for the next one. However, a complete analysis was not conducted until all the interviews had been transcribed.

### Ethics

The study was assessed and approved by the privacy officer of Innlandet Hospital Trust (#113313). Participants received written and oral information about the study and signed a consent form prior to the interviews. It was emphasized that participation was voluntary and that participants could withdraw from the study at any time without stating a reason. During the interviews, the interviewer repeatedly informed the participants that they did not need to talk about sensitive experiences if it made them feel uncomfortable. Two participants were under 16 years of age and consent was therefore obtained from their parents. Before each interview, an agreement was made that the participant’s therapist would be available if the participant needed a follow-up consultation after the interview.

## Results

In accordance with our two research questions, we present the participants’ reflections on their need for therapy and on their daily life functioning in the following.

### Participants’ understanding of their need for therapy in the CAMHS

Only one participant did not express any understanding of why she received therapy at CAMHS. She said:

“*I really don’t know why I’m going to the clinic. I haven’t had much info about it… I don’t really care… I just come along.*” (Felitti et al., 2019).

The others emphasized that they expected therapy partly to help treat their traumatic experiences and partly to focus on coping in their daily lives. The adolescents used the term “trauma,” as well as other psychologically charged terms like “depression” and “anxiety,” but mainly used everyday words. Their ways of mentally working with their traumas involved sorting their experiences, which could be about domestic violence, quarrels between parents or between parents and children, sexual or other kinds of abuse, lack of care, and other difficult situations.

A recurring theme was how parents with mental health problems and alcohol/drug abuse caused traumatic stress and poor daily functioning. One put it this way:

“*I’m going to treatment because my mother drunk a lot of alcohol… and with my father I’ve had…what should I say… lots of problems with communication… kept it to myself… I have a half-brother I haven’t lived with, haven’t talked about that for a very long time. I’ve gotten very anxious and very… especially with people–and I’m depressed and really worn out mentally and physically. After school I just have to go home and sleep… no energy left… and I have a lot of anger that’s been a big problem over the years.*” (Birkeland et al., 2021).

Another theme was how trauma inflicted by parents or other significant adults was not only a past trauma, but a continuous process. One example was about how the parents took the Child Welfare Service to court claiming to get back parental rights for one of the participants. These parents even had told her that they would commit suicide if she did not voluntarily move home to live with them. Her story is an example of how trauma was an ongoing process, and how therapy was an opportunity to deal with this pressure. The participants stated that they needed someone to talk to, an adult person who could understand, which was not the case with many of the parents they referred to. “I cannot handle this on my own” was a typical statement.

On the other hand, being physically or emotionally separated from their parents meant feelings of sadness and grief. There seemed to be an undercurrent of sadness among many of the participants, both related to the damaged relationships with their parents and to the complexity of the traumatic stress and their poor daily functioning.

A recurring theme was hopelessness:

“*I really feel quite a lot of hopelessness. I don’t know how to describe it, but I feel like it’s not going to get any better*.” “*I’m just trying to get through it really… I don’t look forward to the next day…*” (Bellis et al., 2019).

Another theme was avoidance. They said they avoided situations or people when their emotions became too intrusive, and they needed someone to talk to who understood them. The therapy was perceived as a place where they could discuss strategies for coping with situations. Associated with avoidance was the feeling of insecurity, both alone and with others. Several said that they always felt insecure when they were alone at home or when they went out. A typical comment was:

“*I feel really insecure by myself and socially. I can’t be alone at home or go out without feeling insecure. I have a hard time sleeping and being in the dark. I don’t trust many people.*” (Nordanger et al., 2011).

Other prominent themes that the participants associated with their need for therapy were that they could feel insecure in many situations, and feel stressed, depressed, anxious or paralyzed. One of the participants said that she struggled with voices in her head when she was stressed, which made her feel like harming herself. She said:

“*Like I said, it’s the voice in my head, you see… or it’s like if I hang out with a lot of people around me, I get stressed and just want to get away, just want to cut myself on the first thing I see… there’s a whole lot of that, you see…*” (van der Kolk, 2014).

In their efforts to find beneficial strategies for coping with their trauma-related issues and to find relief, CAMHS was seen as important. The data give an example of how CAMHS therapy, which is a specialist level service, was understood in relation to the primary health service contexts and referrals from general practitioners (GPs) as an alternative to psychotropic medication. The following exemplifies this connection between health service levels:

“*Well… it all started with me being very depressed a year and a half ago… a year ago it really started… …then it took six months… a bit more than that maybe… before I started at the clinic… I had a lot of problems with anxiety…I went to see the doctor with my mother… and asked if there was… maybe I could get some medicine that could help…. She said no to that… I mean the doctor… …so then I was supposed to go to the clinic like… and there we soon started talking about things that had happened before and why I was unhappy.*” (Bellis et al., 2019).

It is also possible to see a recurrent theme in the data regarding a dilemma between resigning, “I’m used to it,” or finding a way toward alleviation or coping. For the latter the CAMHS therapy was spoken of as a place for emotional relief where the traumatized adolescents felt that they were noticed and taken care of by safe and secure adults. But, as mentioned above, the material also gives an example of a more indifferent attitude, “I really do not care.”

### Daily life functioning

Participants felt that their daily life was a challenge in several ways. Typical comments were that it was difficult to keep to routines and to feel safe among other people. It was important for them to have something to go to, such as school, and work for a few of them. As mentioned above, their days were marked by mood swings, sadness, hopelessness, indifference, fear and a great deal of frustration. They described being exhausted and some mentioned losing their temper. However, there were also participants who said that they felt pretty good, and that things were better now than before.

Sleeping problems were recurring issues, as well as concentration difficulties at school and with homework. They dealt with this in different ways. Some were motivated for keeping up with their schoolwork, while others resigned or accepted that they were unable to do all the work they saw the others could do. One participant said:

“*It’s very difficult to go to school, difficult to stay in class, to be focused, I often end up having to go home, or I get picked up. Mom picks me up.*” (Hughes et al., 2017).

Many of these adolescents found social contexts difficult. They did not trust people and did not feel safe with others. They all had negative experiences with other people, such as their parents, siblings or others close to them. This was expressed in several ways. One said:

“*I really only trust the friends I had when I was a little girl, before all the stuff happened. I have very good routines, but I’m not very sociable anymore. So, it really made it hard for me to meet other people.*” (Nordanger et al., 2011).

Another participant explained:

“*It (trauma) makes it really difficult to socialize, sleep regular hours, feel safe with other people and your family for example.*” (Hughes et al., 2017).

Different kinds of dilemmas were presented, like one of the adolescents who had chosen to move out of her home because of too much arguing with her parents. She now lived with her boyfriend but was unsure about what to do next, continue living with him or move back home, as she felt she had a better relationship with her father now. The dilemma stressed her, and she explained that she focused on video games as a way of escaping from it.

Routines were mentioned as important for all participants, but many found it difficult to keep to them. Forms of escape or distractions were also mentioned in relation to routines:

“*My routines are pretty bad, I sleep badly at night, I try to find distractions like being with friends, working, being at school, doing my schoolwork.*” (Rueness et al., 2020).

At the same time, some participants said that they felt pretty good and perhaps better now than before. This was not only due to starting therapy, but also changes in their lives. One had moved from her parents’ home to a foster home and felt that life became much easier after that. She said:

“*I find things are much easier… there’s no more… so I’m more serious about school now than I used to be… before I forced myself…. now I go there because I want to. And I feel safer now coming home to my foster parents than my biological ones.*” (Thoma et al., 2021).

The participants had certain common features such as tension, stomach problems, poor sleep and great restlessness. This could be expressed as nausea, dizziness, numbness, trembling when something reminded them of the trauma (trauma triggers), such as smells or seeing something reminiscent of their previous negative experiences. They found it difficult to talk or think about what had happened. They all slept poorly or had nightmares, and never felt that they could relax completely.

Many participants reported bodily sensations when something happened around them. That could make them afraid. They particularly mentioned tension, mostly at school but also at home. Several of them were very frightened in social settings. One became restless and could walk around the room, often his classroom. He pulled loose skin off his fingers when he was restless. He said that he tried to stay awake, because he had seen his father being violent toward his mother and was afraid of falling asleep in case his father came and he did not hear it and was unable to help her.

“*So, it’s more like I can’t fall asleep, because every time I go to bed I have the feeling that my father could show up, because when I sleep I’m quite a deep sleeper and then there’s no chance I can help if something happens.*” (Hughes et al., 2017).

One girl reported becoming tense and stressed when she was afraid. She also had flashbacks when she saw cars that were similar to her parents’ car. She said she was afraid they would take her with them, and then she felt that she just wanted to get away.

All the participants mentioned sleep problems. Most said they slept irregularly and that when they lay down, they felt very uncomfortable, frightened and worried. Many had nightmares; one girl dreamt that she found her father when he had suddenly and unexpectedly died. She also had nightmares about her grandfather’s violence at home.

“*I have a lot of pain in my stomach, it’s very bad, I had to go to the toilet a lot because I’ve been so stressed all the time. I get so tense all the time, a lot of stomachache because of that. And a lot of headaches because I sleep badly. Because I’ve been thinking so much at night.*” (Rueness et al., 2020).

Another participant reacted to the smell of alcohol. It reminded her of her unpredictable mother and the insecurity she felt at home, and she could also have nightmares about things related to her experiences from home. She said:

*“… I never knew what kind of mood my mother was in, if she was kind, happy, angry, in a terrible rage… I never knew… it wore me out.*” (Birkeland et al., 2021).

Their situations were generally characterized by instability, bodily and emotional pain, insecurity, and always being on alert.

## Discussion

The results largely confirm previous findings on the consequences of childhood experiences of trauma (Cruz et al., 2022). However, the present study adds new perspectives on these patients’ understandings and reflections on what help and what treatment they need.

In the first research question, we asked how adolescents with symptoms of developmental trauma described and reflected on their need for therapy in CAMHS.

A prominent finding was that the participants expressed that they needed therapy for symptom alleviation and coping tools, as well as they expressing a need for talking with a safe and reliable adult who understood their situation. Adolescents’ motivation for treatment is not obvious. As far as we have learned, this has rarely been investigated in this patient group. However, studies on, e.g., eating disorders, have found that there may be low expectations for therapy among adolescents (Dittmann and Jensen, 2014). Nevertheless, the present study focused on questions about the participants’ understandings and reflections on why they needed therapy, not on their individual experiences of the actual therapy or the services provided. There were also more girls than boys in this study, and it has been shown that boys are less willing to accept therapy and drop out more often (Berg et al., 2022).

When it comes to our second research question about how these adolescents did describe and reflect on their daily life functioning, the study findings concur with much of what other research shows about the consequences of traumatic experiences (Cruz et al., 2022). Most participants suffered from fatigue and mood swings. An unstable life and poor sleep habits were described, coupled with stress and restlessness. They felt sad, depressed and anxious, as well as hopeless. The data gave examples of dilemmas, ambivalence and mixed loyalties, and demonstrated different strategies of avoidance, distraction, or stress relief.

Negative emotions seemed to be quickly triggered, as seen in other research (Braarud and Nordanger, 2011; Spinazzola et al., 2021). Children with trauma struggle with regulation and recognition of emotions (Walden and Smith, 1997; Pollak and Sinha, 2002). They respond more immediately and strongly to emotional stimuli and need more time to calm down after an emotional reaction (Ebner-Priemer et al., 2007, 2008; Masten et al., 2008; Cruz et al., 2022). Their insecurity makes some participants avoid different settings, as also stated by informants in this study. Avoidance and distraction are common strategies in developmental trauma (Teicher et al., 2016; Felitti et al., 2019).

The fact that the adolescents were able to tell about their experiences is an important finding. The exploratory design made it possible to bring forward daily life examples of what it means to be an adolescent with developmental trauma, which adds to the quantitative research in the field.

An interesting finding regards the path from the GP to CAMHS therapy. For one of the participants in this study, the way the GP had motivated her for other treatment than medication, seemed to have been an important part of her patient narrative. The perspective of cross-level services is important, since for many patients CAMHS therapy is part of a chain of other health and social care service providers such as GPs, school nurses, psychologists, child welfare, and foster homes. Close collaboration across care levels can be of great importance to keep up patients’ motivation for therapy (Danbolt et al., 2016).

### Strengths and limitations of the study

The study is based on eight interviews among adolescents with developmental trauma. It could be argued that the sample size was small, especially given this is a newer area of inquiry, and thus a possible limitation. On the other hand, in qualitative research the information power does not solely depend on the sample size, but on such factors as the quality of the research questions, sample specificity, theoretical anchoring, interview dialog and analysis strategy (Malterud et al., 2016). Qualitative research does not aim at generalizability, but it may be argued that results from this study would be transferable to corresponding patient populations and therapeutic contexts with similar patient groups.

Three of the interviews had to be conducted by telephone due to COVID-19. Body language and facial expressions could therefore not be observed, which may have resulted in a loss of nonverbal information that might otherwise have steered the direction of the interview. The sampling took place on the basis of the therapists’ assessment that the patients met the criteria for inclusion, and the stories told by the participants confirmed this.

## Conclusion and implications

This study has explored how a group of adolescents with developmental trauma described and reflected on their daily life functioning and their need for therapy in CAMHS. For these persons, the symptom picture aligned with what has been described in other studies and diagnostic manuals. A contribution from this study regards the knowledge about these patients’ expectations that therapy can meet their need for symptom alleviation and daily life coping, as well as the information that adolescents with developmental trauma are capable to articulate their experiences and their expectations. Person-centered approaches worked well in this research project, and probably also will in the treatment of adolescents with developmental trauma experiences.

Based on the results, we recommend that adolescents with DT be allowed to express their understandings and treatment expectations at an early stage of their therapy. A person-centered approach where their voices are clearly heard with an emphasis on the therapeutic relationship can increase their autonomy and control over their own lives and treatment.

There is a need for further research on subjective reflections on needs for therapy, motivational factors, and the experienced value of therapy regarding coping tools, symptom alleviation and having a trustworthy adult to talk to. Better targeted interventions will require a deeper exploration of qualitative factors not only regarding such issues as when and where trauma occurs, for how long, and its typical features, but also the patient’s narratives of vulnerabilities, as well as understanding of their situation and their personal, environmental and cultural preconditions for dealing with their situation.

## Data availability statement

The original contributions presented in the study are included in the article/supplementary material, further inquiries can be directed to the corresponding author/s.

## Ethics statement

The studies involving human participants were reviewed and approved by the Privacy Officer of Innlandet Hospital Trust. Written informed consent to participate in this study was provided by the participants’ legal guardian/next of kin. Written informed consent was obtained from the minor(s)’ legal guardian/next of kin for the publication of any potentially identifiable images or data included in this article.

## Author contributions

KO and LD designed the study and made the interview guides. KO collected the data, conducted interviews, and wrote the manuscript. KO, LL, TS, and LD performed the analysis. All authors contributed to the article and approved the submitted version.

## Conflict of interest

The authors declare that the research was conducted in the absence of any commercial or financial relationships that could be construed as a potential conflict of interest.

## Publisher’s note

All claims expressed in this article are solely those of the authors and do not necessarily represent those of their affiliated organizations, or those of the publisher, the editors and the reviewers. Any product that may be evaluated in this article, or claim that may be made by its manufacturer, is not guaranteed or endorsed by the publisher.
